# An Intelligent Sensing Framework for Early Ransomware Detection Using MHSA-LSTM Machine Learning

**DOI:** 10.3390/s26030952

**Published:** 2026-02-02

**Authors:** Abdullah Alqahtani, Mordecai Opoku Ohemeng, Frederick T. Sheldon

**Affiliations:** 1 Department of Computer Science, Najran University, Najran 61441, Saudi Arabia; amjobran@nu.edu.sa; 2 Science and Engineering Research Center, Najran University, Najran 61441, Saudi Arabia; 3 Department of Computer Science, University of Idaho, Moscow, ID 83843, USA

**Keywords:** ransomware, intelligent sensing, innovative sensing technology, Multi-Head Self-Attention (MHSA), Long Short-Term Memory (LSTM), behavioral analysis, cybersecurity

## Abstract

Ransomware represents a critical and evolving cybersecurity threat that often evades traditional defenses during its early stages. We present a novel intelligent sensing framework (ISF) designed for proactive, early-stage ransomware detection, centered on a Multi-Head Self-Attention Long Short-Term Memory (MHSA-LSTM) sensor model. The core innovation of this sensor is its self-attention mechanism, which is augmented to autonomously prioritize the most discriminative behavioral features by incorporating a relevance coefficient derived from information gain (μ), thereby filtering out noise and overcoming data scarcity inherent in initial attack phases. The framework was validated using a comprehensive dataset derived from the dynamic analysis of 39,378 ransomware samples and 9732 benign applications. The MHSA-LSTM sensor achieved superior performance, recording a peak accuracy of 98.4%, a low False Positive Rate (FPR) of 0.089, and an F1 score of 0.972 using an optimized 25-feature set. This performance consistently surpassed established sequence models, including CNN-LSTM and Stacked LSTM, confirming the significant potential of the ISF as a robust and scalable solution for enhancing defenses against modern, stealthy threats. Most significantly, integration of μ as a statistical anchor resulted in a 49% reduction in False Positive Rates (FPRs) compared to standard attention-based models. This addresses the main operational barrier to deploying deep learning sensors in live environments.

## 1. Introduction

In the age of the Internet of Things (IoT), technological progress has heightened specific cybersecurity challenges that hinder seamless integration into daily life and business [1,2]. Among these, the escalating issue of malware, malicious software, designed to breach and disrupt systems, poses a threat to the privacy, integrity, and accessibility of data. Cyber adversaries deploy various types of malware, including Viruses, Trojans, Worms, and, notably, ransomware, to maximize harm [3].

### 1.1. The Escalating Threat of Ransomware

Ransomware, a category of malware, encrypts users’ data and files and requires a ransom for their release [4]. Since its emergence in the late 1980s, ransomware has become a significant menace, impacting a wide range of devices, including personal computers, servers, mobile devices, and IoT devices [5]. Through the creation of ransomware, hackers have brought the concept of extortion into the digital sphere [6].

### 1.2. Global Impact and Economic Ramifications

Ransomware attacks have recently increased dramatically, with financial incentives attracting cybercriminals to develop various strains [6,7]. Not only are individuals targeted but also businesses and governmental institutions [8]. These attacks resulted in cybercriminals making approximately $3 million in 2014 and victims paying approximately $352 million in 2015 to recover their locked data [9]. Additionally, a single county in Indiana spent $220K in recovery efforts from ransomware attacks in 2016 [10]. Beyond the inability to access data, victims face additional damages, including downtime costs, financial losses, and reputational damage. An estimated 623.3 million attacks occurred globally in 2021, with projections reaching 1.5 billion by 2025. The U.S. is the most targeted, followed by the U.K., Canada, and Germany. Businesses can safeguard themselves by implementing strong cybersecurity measures, educating employees about the threat, and having a plan in place. In 2023, the average ransom demand was $170,000, with only 20% of paying businesses retrieving their data. A growing target includes critical infrastructures, emphasizing the serious and escalating threat ransomware poses [7].

### 1.3. Challenges in Early-Stage Ransomware Detection

Crypto-ransomware differs from other types of malware in two main ways: its similarity to benign programs in behavior and the irreversible consequences of its effects [11,12]. By employing system-approved cryptography applications and APIs to target user-related files, crypto-ransomware mimics benign program behavior [13,14]. Moreover, the use of cryptography renders targeted files inaccessible even after the offending crypto-ransomware has been detected and removed [15,16]. Once ransomware encrypts a target, regaining access without the decryption key proves difficult. This irreversibility necessitates early detection to effectively combat ransomware [17]. However, early detection is challenging. Several studies have been carried out to address this issue, resulting in proposed early detection solutions [7,17,18,19]. These solutions leverage sensing technology by collecting data from the initial stages of known crypto-ransomware attacks and using machine learning models to detect new, unknown attacks. Yet, the main obstacle to effective early detection is the absence of sufficient information at the onset of the attack [19]. Current sensing solutions assume the data necessary for detection is complete and ready to use at the time of detection, which is not the case for early detection while the attack is in progress and the data are incomplete [7]. Therefore, the initial data lacks enough attack patterns, leading to the creation of weak classifiers [9].

### 1.4. Existing Threshold and Behavioral Analysis Approaches

Various ransomware detection proposals combine specific parameters to measure software dynamic activity and characterize process activity, thus allowing a process to be labeled as benign or ransomware based on certain thresholds. Examples include the framework developed by [20], which acts as a sensor to measure disk access actions with primary and secondary indicators, and in [21], an investigation of Solid State Disks behavior. The study by [22] used multiple input parameters related to disk access actions as a form of sensing, while [23] used a honeypot where file changes were monitored. The study conducted in [24] focused specifically on image files, while a different approach in [25] measured network traffic between an infected computer and shared volumes. Other proposals, like those from [26,27,28], targeted ransomware detection in the Android cell phone operating system, using various sensing techniques such as text detection, file access monitoring, and written file monitoring.

### 1.5. Machine Learning Techniques for Ransomware Identification

Machine learning (ML) techniques have become essential for identifying complex patterns across various domains, ranging from cybersecurity to bioinformatics. For instance, ML has been leveraged to predict bacterial population dynamics to combat antimicrobial resistance [29], demonstrating the versatility of these algorithms in identifying malicious growth patterns before they become uncontrollable. Similarly, in the digital realm, ref. [30] used supervised learning algorithms with Microsoft Windows kernel modules as a form of intelligent sensing, and the EldeRan [31] adoption of a supervised regularized logistic regression ML algorithm. Reference [32] implemented a Support Vector Machine (SVM) classifier, while [33] employed the V-detector, a negative selection algorithm. Other approaches include [34] the usage of Windows API calls, ref. [35] observations of hardware performance counters, and [36] analysis of file system traversal action. Some proposals employed a hybrid approach, combining ML detection, threshold-based detection, and ad hoc mechanisms, such as the method proposed in [37]. Other proposals, like refs. [38,39], used ML only when a certain number of features were detected. ML algorithms have also been applied to crypto-ransomware detection in the Android operating system, as seen in R-PackDroid [40,41] ransomware detection methods.

### 1.6. Deep Learning Solutions and the Need for Attentive Modeling

Several studies tackled the early detection of ransomware, some of which used deep learning. An adaptive sensing system is required to deal with the development of variants of ransomware [42]. Some studies tried to develop an adaptive framework with limitations. An adaptive ransomware detection framework was proposed by [43], which combined supervised and unsupervised approaches to achieve adaptiveness. It was an anti-obfuscation model that extracted features from runtime data, functioning as an intelligent sensor. This semi-supervised model was implemented using deep learning. Long Short-Term Memory (LSTM), a deep learning algorithm, was used by several malware detection studies. The study conducted by [18] used a CNN-LSTM model to overcome challenges in malware detection, including human feature, building inefficiency and limitations of existing algorithms. By stacking CNN and LSTM techniques, they created a deep neural network ensemble capable of detecting advanced malware without feature engineering. Similarly, ref. [44] is a solution based on a stacked Long Short-Term Memory (LSTM) that incorporates pre-training as a regularization approach to prevent arbitrary network initialization. It leverages both global and short-term input dependencies, reducing the need for full-length malware behavioral data. Therefore, it is suitable for early detection when data is insufficient.

### 1.7. Architectural Innovations and Differentiations

However, these studies treat all input features equally, regardless of their relevance and variance. The less relevant features make the sensor model learn more noise, especially when the data does not contain enough attack patterns. This leads to less accuracy and high false alarms. To this end, our study overcomes this issue by incorporating improved self-attention techniques into the LSTM training. Intuitively, adding the self-attention mechanism allows the LSTM-based ransomware sensor to focus on the most relevant parts of the input sequence when making predictions. This helps the intelligent sensor to use important attack patterns, which compensates for the need to capture long-term dependencies.

Unlike [45,46,47], which allow the attention mechanism to converge solely through backpropagation, our framework implements a hybrid weighting strategy. By incorporating information gain (μ), we provide a deterministic prior that stabilizes the framework during the data-scarce early phases of ransomware execution.

The contribution of this paper is threefold:

1. An information gain-based self-attention technique that provides a deterministic prior to the model. This specifically mitigates the Signal-to-Noise problem in early-stage detection, where benign system activity often mimics ransomware entropy, thereby achieving a near-zero False Positive Rate essential for minimizing user disruption.
2. This technique was incorporated into the LSTM model, which boosts the ability of the ransomware detection sensor to perceive the relevant attack patterns more accurately.
3. An extensive experimental evaluation was conducted to confirm the improvement that the proposed intelligent sensing framework achieved for ransomware early detection compared to existing solutions.

### 1.8. Paper Organization

The rest of this paper is organized as follows. In Section 2, related works were explored. Section 3 describes the methodology and proposed techniques. Section 4 presents and discusses the experimental results and comparison with related models. The paper ends with a conclusion section in Section 5 that revisits the work done and provides suggestions for future research.

## 2. Related Works

The limitations of traditional signature-based detection have necessitated a shift toward advanced, behavioral-based detection methods, which function as intelligent sensors to analyze application execution. This section reviews existing ransomware detection techniques, categorized by their sensing mechanisms, to contextualize the necessity of our proposed attentive deep learning framework.

### 2.1. Static and Threshold-Based Sensing Techniques

Early behavioral solutions moved toward identifying malware through general characteristics rather than unique signatures. These methods, functioning as passive sensors, primarily involve static analysis or simple threshold monitoring.

Techniques such as searching for encryption primitives or monitoring possible encryption keys are often resource-intensive and prone to high False Positive Rates (FPRs).

A common tripwire approach involves closely monitoring decoy or canary files for unauthorized changes. While simple, this method is vulnerable to advanced ransomware strains that encrypt files selectively to evade detection.

Other proposals combine specific parameters, often related to disk access actions, to characterize process activity [20,21,22]. While these solutions provide a quick classification based on defined thresholds, they lack the adaptability required to distinguish novel ransomware variants from complex benign processes [11,12].

### 2.2. Active, Network-Based, and Hybrid Sensing Frameworks

More comprehensive detection strategies involve active behavioral sensing through continuous system monitoring and network analysis, often requiring high overhead.

Frameworks monitor system calls (API) and disk access by user programs [22,25]. The study in [25] used a Pre-Encryption Detection Algorithm (PEDA) combining signature matching and Machine Learning (ML) to target the critical pre-encryption stage. While effective in theory, the overhead of constant system monitoring leads to high CPU usage and can generate unacceptable false positives, significantly impacting user experience.

Solutions analyzing network traffic [25] offer another form of sensing but can be bypassed if the ransomware employs local encryption keys or uses sophisticated cloaking techniques to hide its command and control communication.

Advanced frameworks have integrated multiple sensing methods. For example, a framework combined incremental bagging (iBagging) and enhanced semi-random subspace selection (ESRS) to build subsets mirroring the evolution of an attack [9]. Others used ML on hardware performance counters (HPCs) [11]. The common limitation among these hybrid approaches is their reliance on manual feature engineering or complex ad hoc mechanisms [37].

### 2.3. Deep Learning Frameworks for Sequence Sensing

The constant evolution and surge in the sophistication of ransomware, particularly the rise in Ransomware-as-a-Service (RaaS) and the manifold attack increase noted in [48], demand adaptive, sequence-aware deep learning (DL) models. These models function as intelligent sensors, capturing complex temporal dependencies in behavioral data.

The necessity for precision in sensing is not unique to cybersecurity: in autonomous vehicle systems, lateral error feedback analysis is utilized to enhance control and stability [49]. Just as autonomous systems require feedback to correct trajectory errors, ransomware detection frameworks require attentive modeling to correct the model’s focus toward high-entropy features. This transition toward more sophisticated, feedback-driven sensing is critical in capturing both short- and long-term dependencies that lead to advanced RNN structures. Studies combined LSTM and Gated Recurrent Unit (GRU) networks for Android malware detection [50] and integrated LSTM into larger, comprehensive frameworks for temporal anomaly detection in IoT environments [51].

Beyond basic architecture, some DL solutions address security in high-risk sectors like the Internet of Things (IoT) [52]. For instance, the DLEX-IMD approach uses a deep learning ensemble for IoT malware detection and incorporates Explainable AI (LIME) to justify the model’s reliability and enhance interpretability [53].

More recent work has introduced attention mechanisms to enhance the focus of DL sensors. The MHARNN-EGTOCRD approach in [46] and the MDMIoV-DLXAI framework in [47] both implemented Multi-Head Attention (MHA) hybrid classifiers (MHA-LSTM, BiLSTM-MHSA), demonstrating the trend toward using explicit weighting mechanisms to capture salient features. These works further enhance performance by incorporating sophisticated optimization algorithms primarily for hyperparameter tuning.

### 2.4. Gap Identification: Feature Relevance and Attentive Weighting

Despite the advancement to attention-based DL, a significant research gap persists concerning the dynamic utilization of feature relevance during the classification process.

While advanced frameworks have been developed for dynamic feature selection, such as the Incremental Mutual Information Selection (IMIS) [54] or the Dung Beetle Optimization (DBO) method [46], these approaches perform selection before the final classification. This means the feature set is static during the time-step analysis, and the attention mechanism lacks an external, statistically grounded prior.

The core limitation across existing attention-based solutions [44,45,46,47] is that they still treat the raw input features equally when initiating the self-attention mechanism. In the critical early stages of an attack, where data is incomplete and attack patterns are scarce, this uniform weighting causes sensor models to learn noise from features that have low inherent predictive power (low information gain). This leads directly to two critical performance drawbacks, a decrease in accuracy and unacceptably high False Positive Rates (FPRs), as highlighted by multiple surveys [52,55].

To mitigate this, our framework integrates statistical relevance (μ) directly into the attention mechanism, allowing it to autonomously prioritize and weight features based on their intrinsic predictive value, which no prior work has demonstrated.

## 3. The Methodology

This section details the development and validation of the proposed intelligent sensing framework (ISF). We first describe the novel Multi-Head Self-Attention Long Short-Term Memory (MHSA-LSTM) sensor architecture, highlighting the integration of the information gain coefficient (μ) as a feature-prioritization mechanism. We then specify the rigorous procedures for data acquisition, preprocessing, and model optimization, and the metrics used to evaluate performance.

### 3.1. Multi-Head Self-Attention LSTM (MHSA-LSTM) for Ransomware Detection

**Figure 1:**
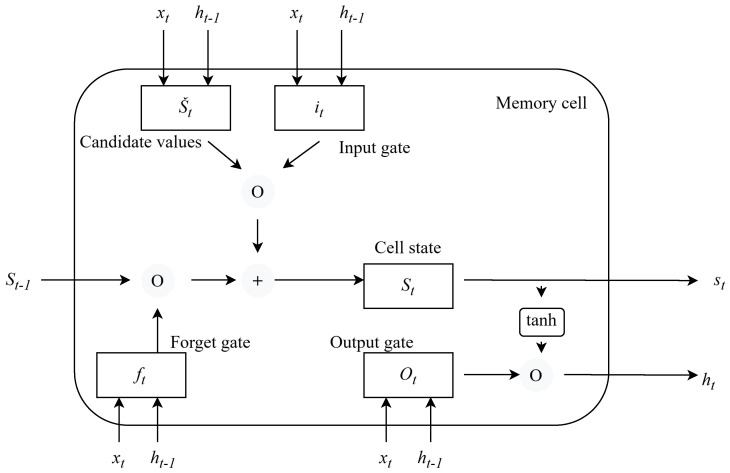
Schematic diagram of the Multi-Head Self-Attention LSTM framework.

The MHSA-LSTM framework is engineered as an intelligent sensor to analyze the time-series behavioral data of applications. The overall architecture integrates two main components: the LSTM Core for sequence processing and the Enhanced Multi-Head Self-Attention mechanism for dynamic feature prioritization. The foundational unit for sequence processing is the LSTM memory cell, whose detailed internal architecture is visualized in Figure 1.

The cell manages its state ($S_{t}$) using three distinct mechanisms, known as gates, which are essentially neural networks that output values between 0 and 1 via the sigmoid function (σ).

The Forget Gate ($f_{t}$) determines which information from the previous Cell State ($S_{t-1}$) should be discarded or forgotten. It looks at the current input ($x_{t}$) and the previous hidden state ($h_{t-1}$) and outputs a number between 0 (complete forget) and 1 (complete retain) for each value in $S_{t-1}$. The Forget Gate’s output is multiplied (using the circle with an ‘O’ for element-wise multiplication) by the previous Cell State ($S_{t-1}$) before being passed to the sum operation.

The Input Gate ($i_{t}$) and Candidate Values (${\tilde{S}}_{t}$). This pair works together to determine what new information will be stored in the cell state.

Input Gate ($i_{t}$): Decides which values in the Candidate Values (${\tilde{S}}_{t}$) will be updated. It acts as a filter, scaling the importance of the new information. It uses the sigmoid function (σ).

Candidate Value (${\tilde{S}}_{t}$): Creates a vector of potential new values that could be added to the state. It uses the hyperbolic tangent function (tanh) to scale the potential new values between −1 and 1. The output of $i_{t}$ is multiplied (element-wise) with ${\tilde{S}}_{t}$.

Cell State ($S_{t}$): The central horizontal line, which represents the direct path of information flow. This is the core of the LSTM, where the new Cell State ($S_{t}$) is computed. It involves an element-wise addition (indicated by the circle with a ‘+’) of two components, the old state ($S_{t-1}$) scaled by the Forget Gate ($f_{t}$), and the new candidate information (${\tilde{S}}_{t}$) scaled by the Input Gate ($i_{t}$). This additive interaction is what allows information to persist across many time steps, solving the vanishing gradient problem inherent in simple RNNs.

The Output Gate ($O_{t}$) and Hidden State ($h_{t}$). This final mechanism determines the cell’s output for the current time step.

Output Gate ($O_{t}$): Selects which parts of the final Cell State ($S_{t}$) will be exposed as the final output. It uses the sigmoid function (σ).

Hidden State ($h_{t}$): The final output of the cell. The final Cell State ($S_{t}$) is first passed through a tanh function to scale the values, and then multiplied (element-wise) by the output of the Output Gate ($O_{t}$). This resulting vector is the Hidden State ($h_{t}$), which serves as the output for this time step and the input for the next. A summary of the definitions of the notations used in the MHSA-LSTM framework is given in Table 1.

#### 3.1.1. The Long Short-Term Memory (LSTM) Core

The LSTM is employed as the foundational component of our sensor, being a specialized Recurrent Neural Network (RNN) variant engineered to effectively model long-term temporal dependencies within the host’s behavioral data (API call sequences). It overcomes the vanishing gradient problem inherent in standard RNNs by using a sophisticated system of internal gates to meticulously regulate the flow of information into the Cell State ($S_{t}$), which serves as the memory pathway.

The forget gate is responsible for deciding which information from the previous Cell State ($S_{t-1}$) is no longer relevant to the current behavioral context and should be discarded.

(1) $$f_{t}=\sigma \left(W_{f_{x}}x_{t}+W_{fh}h_{t-1}+b_{f}\right),$$

where $x_{t}$ is the current input vector (behavioral features) at time *t*, $h_{t-1}$ is the hidden state (output) from the previous time step (t−1), $W_{f_{x}},\;W_{fh},\;b_{f}$ are the weight matrices and bias vector to be learned respectively, and σ (sigmoid function) scales the output $f_{t}$ between 0 (completely forget) and 1 (completely remember).

The input gate ($i_{t}$) and candidate cell state (${\tilde{S}}_{t}$): This phase determines what new information from the current input ($x_{t}$) will be stored in the cell state. This involves two parallel calculations:

Candidate values (${\tilde{S}}_{t}$): Potential new information, scaled by the hyperbolic tangent function (tanh) to the range [−1, 1].

(2) $${\tilde{S}}_{t}=\tanh\left(W_{\tilde{s_{x}}}x_{t}+W_{\tilde{s_{h}}}h_{t-1}+b_{\tilde{s}}\right).$$

Input gate activation ($i_{t}$): Determines the extent to which the candidate values will be used, scaled by a sigmoid between 0 and 1.

(3) $$i_{t}=\sigma \left(W_{i_{x}}x_{t}+W_{i_{h}}h_{t-1}+b_{i}\right).$$

Updating the cell state ($S_{t}$): The new cell state $S_{t}$ is computed by a combination of the previous state, filtered by the forget gate, and the new candidate information, filtered by the input gate. This additive interaction is the core mechanism allowing information to persist across time steps.

(4) $$S_{t}=f_{t}\times S_{t-1}+i_{t}\times \tilde{S_{t}}.$$

The output gate ($O_{t}$) and hidden state ($h_{t}$): The output gate controls what part of the new cell state is exposed as the final output (hidden state) $h_{t}$ for the current time step.

Output gate activation ($O_{t}$): Uses the sigmoid to select relevant parts of the information.

(5) $$O_{t}=\sigma \left(W_{O_{x}}x_{t}+W_{o_{h}}h_{t-1}+b_{O}\right).$$

New hidden state ($h_{t}$): The output of the LSTM cell.

(6) $$h_{t}=O_{t}\times \tanh\left(S_{t}\right).$$

#### 3.1.2. Enhanced Multi-Head Self-Attention Mechanism with Information-Theoretic Guidance

The proposed MHSA mechanism processes the output sequence from the LSTM core ($O_{all}$) to dynamically assign weights, enabling the sensor to prioritize salient features. While traditional attention models rely on stochastic convergence through backpropagation, our framework transitions to an information-theoretic guided paradigm. This addresses the inability of standard models to distinguish discriminative signals from behavioral noise when sequences are truncated during early-stage execution.

##### Theoretical Distinction and μ Dimensionality

Following the principles of additive attention [56], the LSTM outputs define the Queries (*Q*), Keys (*K*), and Values (*V*). In our framework, we introduce a deterministic prior via the relevance vector μ.

Let *F* be the number of features and *L* be the sequence length. We define $\mu \in R^{1\times F}$ as a global relevance vector where each element is the pre-calculated information gain (IG) of a feature.

Although μ is fixed during the inference phase, its role is to provide a deterministic anchor for the attention heads. This choice is preferable to purely learnable weights in early-stage detection. To align with the framework’s internal latent space, μ is projected via a linear transformation to $\mu^{\prime }\in R^{1\times d_{model}}$. Although μ is fixed during inference, it provides a deterministic anchor for the attention heads. To modulate the attention scores, $\mu^{\prime }$ is applied as a weight vector across the projected Query and Key representations. This ensures the framework suppresses noisy system calls, regardless of their temporal position, by anchoring focus on dimensions with high mathematical discriminative power.

### 3.2. Feature Discretization

All continuous features in the behavioral dataset were discretized using equal-width binning. For a continuous feature *x*, the range $\left[x_{\min},x_{\max}\right]$ was divided into *k* discrete bins (k=10 in this study). Each continuous value *v* was mapped to a bin index *b* according to

(7) $$b=\min\left(\left\lfloor \frac{v-x_{\min}}{w}\right\rfloor ,k-1\right),\;w=\frac{x_{\max}-x_{\min}}{k}.$$

This transformation converts the continuous telemetry data into a discrete distribution, allowing for the stable calculation of the μ coefficient. This ensures that the global relevance score captures the underlying information state of the feature without the noise inherent in raw continuous values.

#### Implementation and Tensor-Wise Injection

The process is split into *n* heads. For each head *i*, the projections are defined as

(8) $$K_{i}=O_{all}W_{i,k}+b_{i,k},\;V_{i}=O_{all}W_{i,v}+b_{i,v},\;Q_{i}=O_{last}W_{i,q}+b_{i,q},$$

where $Q_{i},\;K_{i},\;V_{i}$ have the shape $\left(B,L,d_{k}\right)$, where *B* is the batch size. The core innovation is the injection of μ as a relevance multiplier.

The μ coefficient is calculated based on the expected reduction in entropy (*E*) of class labels (*D*) provided by feature *x*

(9) $$\mu =E\left(D\right)-\sum_{i=0}^{m-1}p_{i}E\left(D|x=x_{i}\right).$$

The weighted attention score $s_{i}$ is then calculated by applying $\mu^{\prime }$ to the dot-product attention. To ensure dimensional consistency, $\mu^{\prime }$ is broadcast across the batch dimension (*B*) and sequence length (*L*). μ quantifies the reduction in entropy when the feature is observed in its discretized state. By calculating μ on binned data, we avoid the sensitivity to minor fluctuations in continuous values, ensuring the intelligent sensor prioritizes features that show a distinct, categorical shift between benign and malicious execution paths. The final attention score is

(10) $$s_{i}=\mathrm{softmax}\left(\frac{Q_{i}K_{i}^{T}}{\sqrt{d_{k}}}\right)⊙\mu^{\prime },$$

where ⊙ denotes the element-wise Hadamard product. Here, $s_{i}\in R^{B,L,L}$ (for self-attention) or $R^{B,1,L}$ (when querying with $O_{last}$). This scaling provides energy sharpening. Even if an attack pattern is truncated, its high intrinsic predictive power (encoded in μ) forces the framework to prioritize it, preventing attention diffusion across benign background processes.

The weighted scores are applied to the value vectors to produce context-aware vectors, which are concatenated into the final context vector (CV)

(11) $$context_{i}=s_{i}V_{i},\;CV=\mathrm{Concat}\left(context_{1},\ldots ,context_{n}\right)W^{O}$$

The resulting $CV\in R^{B,d_{model}}$ is passed through a fully connected layer with ReLU activation and a softmax layer for binary classification.

### 3.3. Model Training and Data Preparation

The training of the MHSA-LSTM sensor was structured around a formalized pipeline encompassing rigorous data preparation, hyperparameter optimization, and metric-driven validation.

#### 3.3.1. Dataset Acquisition and Dynamic Analysis

The foundation of our intelligent sensor relies on a comprehensive, behaviorally rich dataset captured through controlled dynamic execution.

The dataset comprises 39,378 ransomware samples (including prominent families such as CryptoWall, Petya, and WannaCry, sourced from Virusshare) and 9732 benign applications (sourced from informer.com).

All executable files were subjected to dynamic execution within the Cuckoo Sandbox, an industry-standard, open-source virtual platform designed for isolated malware inspection. The sandbox meticulously recorded runtime activities, focusing primarily on sequences of API calls, network communication logs, and file system interactions, outputting detailed JSON reports. This sequential runtime data forms the basis of the time-series features fed into the LSTM core.

To ensure the time-series data accurately reflected isolated execution and prevented cumulative behavioral contamination, the virtual guest machine was reset to a pristine condition after the execution of every single sample. This strict protocol is essential for generating reliable, non-compromised training features.

To ensure the integrity of the behavioral features, a multi-stage curation pipeline was implemented (Table 2). Ransomware samples were validated via VirusTotal; a sample was only included if it was flagged as ransomware by at least 10 major antivirus engines. Benign samples from Informer.com were subjected to the same screening to exclude Adware or Potentially Unwanted Programs (PUPs), ensuring a clean baseline for legitimate system activity.

To prevent data leakage, we performed MD5 hashing to remove duplicate binaries. The dataset covers a temporal window from 2018 to 2024, ensuring the framework is exposed to both legacy encryption methods and modern, sophisticated evasion techniques.

#### 3.3.2. Data Preparation and Feature Engineering

The raw behavioral data extracted from dynamic analysis reports were subjected to rigorous preparation to ensure the construction of a high-quality, time-series numerical feature matrix while actively preserving the chronological dependencies crucial for the LSTM core.

A statistical filter based on the Z-score ($Z_{i}$) was applied to identify and remove statistical outliers ($x_{i}$) to prevent corruption of the training process.

(12) $$Z_{i}=\frac{x_{i}-\bar{x}}{\sigma }.$$

Data points where $|Z_{i}|>3$ were typically removed.

All feature attributes were scaled to the interval [0, 1]. This measure mitigates the bias in weight updates caused by features with disparate value ranges.

(13) $$x_{\mathrm{norm}}=\frac{x-x_{\min}}{x_{\max}-x_{\min}}.$$

A compact, non-redundant feature set was selected using the NHRCU-MIFS strategy [35], which is optimized for maximum relevance to the binary class label.

The core of the selection process involves calculating the information gain (μ), which serves as the quantitative relevance score. μ measures the expected reduction in the dataset’s entropy (*E*), the measure of impurity or randomness provided by an attribute *x*.

(14) $$E\left(D\right)=-\sum_{j=1}^{C}P\left(c_{j}\right)\log_{2}P\left(c_{j}\right),\;$$

where $P(c_{j})$ is the probability of an instance belonging to class $c_{j}$.

The μ coefficient in Equation (9), calculated as the information gain, quantifies the feature’s power to discriminate between the Ransomware and Benign classes.

This pre-calculated μ value is stored and later injected into the Multi-Head Self-Attention mechanism, to act as a relevance score multiplier.

To transform raw dynamic execution logs into a structured temporal format, we map the chronological API sequences into a tensor of shape (B,200,25), where *B* is the batch size and T=200 is the fixed sequence length. For execution traces shorter than 200 steps, we apply post-padding with zero values. To prevent the MHSA-LSTM from attending to these non-informative steps, a Boolean masking layer is utilized. This layer assigns a value of −∞ to the attention scores of padded indices prior to the softmax operation, ensuring that the resulting context vector (CV) is derived solely from valid behavioral data.

Following the NHRCU-MIFS strategy, a final set of 25 features was isolated based on their information gain (μ) coefficients. These features represent the behavior of ransomware, spanning critical domains such as file manipulation, registry persistence, and shadow copy deletion. The complete list of features and their corresponding malicious intent is detailed in Table 3.

#### 3.3.3. Model Training and Hyperparameter Optimization

The refined sequence data is fed into the deep learning architecture for training, incorporating the novel μ-weighted attention mechanism.

Time-series features are processed by the LSTM layers to capture spatial and temporal patterns across the execution sequence.

All hidden states ($O_{all}$) are passed to the Multi-Head Self-Attention (MHSA) layer. The μ coefficient directly modulates the attention scores ($s_{i}$) (Equation (10)), ensuring that the sensor signifies the relevant attack patterns even when the data sequence is short or incomplete during early detection.

The resulting context vector (CV) is activated via a Rectified Linear Unit (ReLU), passed to a fully connected layer with 50 neurons, and culminates in a softmax output layer for binary classification (Ransomware or Benign).

#### 3.3.4. Optimization

The framework was trained by minimizing the Binary Cross-Entropy Loss (L), a standard objective for binary classification tasks:

(15) $$L=-\frac{1}{N}\sum_{i=1}^{N}\left[y_{i}\log\left({\hat{y}}_{i}\right)+\left(1-y_{i}\right)\log\left(1-{\hat{y}}_{i}\right)\right].$$

Dropout layers were utilized to mitigate overfitting, and Batch Normalization was applied to hidden layers to stabilize inputs and accelerate training convergence.

### 3.4. Experimental Environment and Evaluation Metrics

The framework’s implementation used Python 3.11 alongside the deep learning frameworks TensorFlow and Keras, supported by Scikit Learn and Numpy. All processing was conducted on a machine featuring an Intel^®^ Core™ i7-4790 CPU @ 3.60 GHz and 16 GB of RAM.

To ensure statistical significance and assess robustness, we employed 10-fold group cross-validation. Unlike standard cross-validation, the group-based approach ensures that all samples belonging to a specific ransomware family are kept together, a family is never split between training and testing sets within any fold. This prevents data leakage and ensures the reported mean (μ) and standard deviation (σ) reflect the framework’s ability to generalize across different malicious behaviors. Furthermore, to simulate real-world deployment, a temporal constraint was implemented where samples from the 2023–2024 era (including advanced RaaS variants like LockBit 3.0 and BlackCat/ALPHV) were reserved strictly for the independent test set. This ensures the framework is evaluated on its ability to detect modern evasion techniques and zero-day behaviors that were not present in the earlier training data (2018–2022). Samples collected in the final quarter of the dataset were reserved strictly for an independent test set to account for concept drift and evaluate the detection of chronologically newer zero-day variants. This dual-validation strategy ensures the framework learns generalized patterns, such as encryption entropy and shadow copy deletion, rather than overfitting to family-specific API signatures. The search spaces for each framework are detailed in Table 4. By keeping the feature set and tuning budget constant, the performance delta observed directly quantifies the value-add of coupling (μ) with MHSA-LSTM.

In our implementation, μ is implemented as a non-trainable weight layer. This ensures the information-theoretic prior remains fixed based on the global dataset statistics, while the LSTM and MHSA weights ($W_{Q},W_{K},W_{V}$) adapt to the temporal nuances of the specific sequence.

#### Performance Metrics

The framework’s efficacy was evaluated using four standard security and classification metrics based on the True Positive (TP), True Negative (TN), False Positive (FP), and False Negative (FN) counts.

Accuracy (ACC): The proportion of correct predictions (both True Positive and True Negative) out of all cases.

(16) $$ACC=\frac{TP+TN}{TP+TN+FP+FN}.$$

False Positive Rate (FPR): The rate at which benign instances are incorrectly classified as ransomware. Minimizing FPR is critical for any security solution to prevent excessive false alarms.

(17) $$FPR=\frac{FP}{TN+FP}.$$

Detection Rate (DR)/Recall: The proportion of actual ransomware samples that were correctly identified (True Positive). This measures the sensor’s ability to catch threats.

(18) $$DR=\frac{TP}{TP+FN}.$$

F1 score (F1): The harmonic mean of precision and recall. It provides a balanced measure, especially useful when class distribution is uneven.

(19) $$F1=\frac{TP}{TP+0.5\times (FP+FN)}.$$

## 4. Results and Discussion

This section presents the empirical validation of the proposed Multi-Head Self-Attention Long Short-Term Memory (MHSA-LSTM) intelligent sensing framework. We first analyze the sensor’s performance across various feature scales and then provide a comprehensive comparative analysis against established deep learning models, focusing on the influence of the novel self-attention mechanism. The performance was evaluated using accuracy (ACC), False Positive Rate (FPR), Detection Rate (DR), and F1 score (F1).

### 4.1. Feature Optimization and Sensor Performance Analysis

The initial assessment focused on determining the optimal complexity of the input data stream by measuring the sensor’s performance metrics across a range of feature counts, as detailed in Table 5.

As demonstrated in Table 6, the proposed MHSA-LSTM consistently achieves an accuracy of 98.4%±0.4 and an F1 score of 97.2%±0.5. A critical observation is the trend in standard deviation across the architectures. While the baseline models (CNN-LSTM and Stacked LSTM) exhibit higher variance (up to ±1.4%), the MHSA-LSTM maintains a significantly tighter distribution (±0.4%). This suggests that the framework’s performance is not a result of favorable data partitioning, but rather the architectural stability provided by the μ-weighted attention mechanism. By anchoring the self-attention heads with deterministic information gain (μ), the framework effectively filters out the stochastic noise of benign background processes that often cause performance fluctuations in standard models. This leads to a more robust and reproducible detection capability, ensuring high-confidence sensing even when the framework encounters varying execution environments within the 10-fold group cross-validation.

With just five highly informative features, the sensor already achieves a strong ACC of 0.956 and a high F1 score of 0.964. As the count increased to 15, the FPR significantly decreased to 0.108, demonstrating the improved specificity gained from a more refined input set.

The framework reached its peak in sensing performance metrics with 25 features. At this point, it recorded the highest accuracy (0.984) and the lowest False Positive Rate (0.089). This highlights the optimal trade-off between input data quantity and quality, underscoring the effectiveness of the feature selection technique.

As the feature count increased beyond 25, the sensor’s performance began to slightly decline: the ACC dropped to 0.970 at 30 features and 0.955 at 50 features. This crucial observation suggests that the inclusion of less relevant or redundant data introduces noise, despite the internal filtering capability of the self-attention mechanism. It confirms that an optimal data limit exists for effective and precise sensing in this context.

The consistently high performance, even with minimal features, is attributed to the enhanced self-attention mechanism. By re-weighting input features based on their calculated information gain (μ), the sensor maintains focus on the most discriminative behavioral patterns while effectively filtering out less significant inputs.

### 4.2. Comparative Analysis with Baseline Models

To benchmark the effectiveness of the proposed architecture, the MHSA-LSTM sensor was compared against three established deep learning models for sequence analysis: CNN-LSTM, Stacked LSTM, and ARI-LSTM. These baseline specifically represent the three fundamental paradigms of behavioral sequence modeling, spatial pattern recognition, deep temporal memory, and adaptive rate integration. We contend that these architectures provide a more rigorous stress test for early-stage detection than Transformers, which often require significantly larger datasets and higher computational overhead to converge. To ensure a fair comparison, all baselines were trained on the identical 25-feature set and subjected to a standardized grid search for hyperparameter optimization (tuning learning rates from $1\times 10^{-4}$ to $1\times 10^{-2}$ and dropout from 0.2 to 0.3). This ensures that the observed performance uplift in the ISF is a direct result of the information-theoretic μ-guidance rather than disproportionate tuning or hardware advantages.

**Figure 2:**
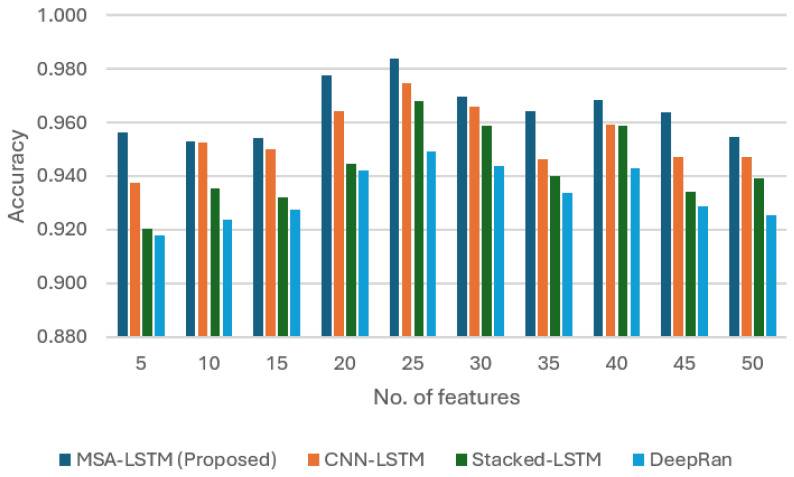
The plot of MHSA-LSTM accuracy rate as compared to other models.

As illustrated in Figure 2, the MHSA-LSTM sensor demonstrated superior and more robust performance in terms of overall accuracy across all feature counts. The MHSA-LSTM achieved a peak accuracy of 0.984 (at 25 features), consistently outperforming the baseline models, whose accuracy was visibly lower and more prone to fluctuation as the feature count increased. This sustained lead highlights the MHSA-LSTM’s exceptional ability to handle the increasing complexity of its input while maintaining precision.

**Figure 3:**
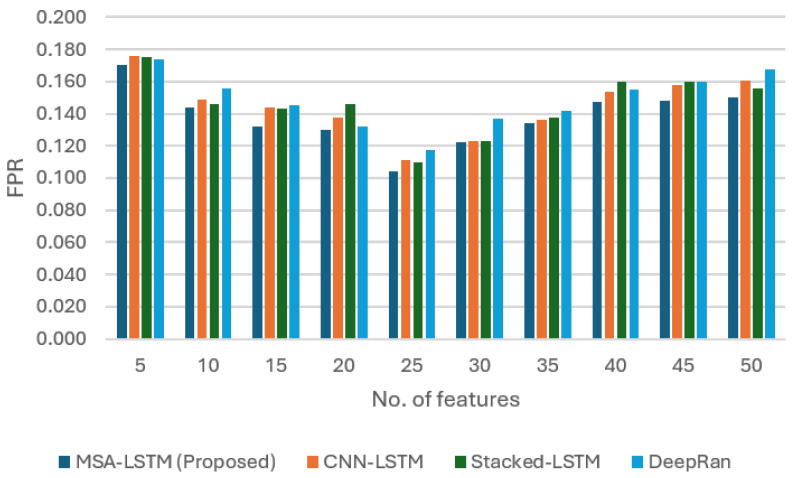
The plot of the MHSA-LSTM sensor performance in reducing the false positive rate (FPR) compared to the other models.

False Positive Reduction: A critical measure for operational security is minimizing the False Positive Rate (FPR), as a low FPR ensures a viable system by limiting false alarms. As shown in Figure 3, the MHSA-LSTM exhibited a clear advantage in controlling the FPR. The MHSA-LSTM recorded its lowest FPR of 0.089 (at 25 features), proving significantly more effective at correctly distinguishing between malicious and benign behaviors than the competing models. This superior specificity is directly linked to the μ-weighted attention coefficient, which enhances the sensor’s ability to focus only on highly relevant, predictive features, reducing sensitivity to benign noise. While standard deep learning architectures like CNN-LSTM and Stacked LSTM achieve high accuracy, they often suffer from stochastic over-sensitivity, misclassifying legitimate encryption or compression as malicious. Our results show that by anchoring the attention mechanism with μ, the ISF filters out these benign coincidences. The drop in FPR from the baseline models to our MHSA-LSTM (from ≈0.18 to 0.089) represents the transition from a research prototype to a viable cybersecurity sensor.

**Figure 4:**
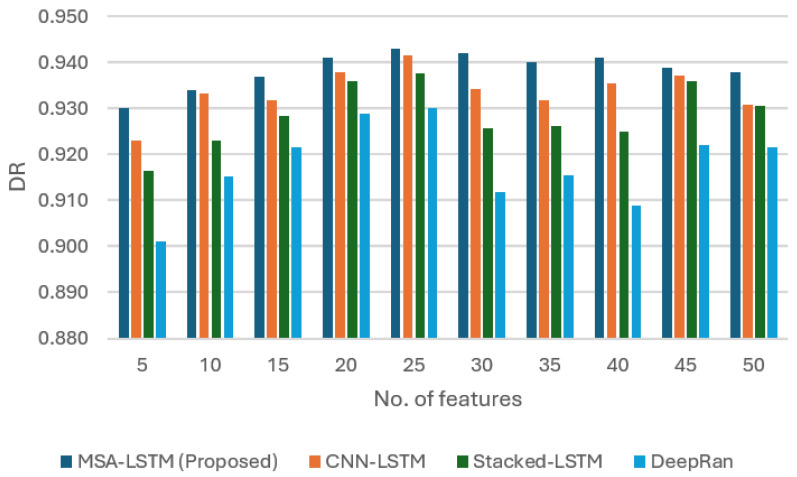
The plot of MHSA-LSTM compared to existing ransomware detection models (CNN-LSTM, Stacked LSTM, and ARO-LSTM).

Detection Rate (DR): The sensor reached a high DR of 0.952 at its peak, confirming its strong early detection capabilities and ability to correctly classify threats (high recall). This can be seen in Figure 4.

**Figure 5:**
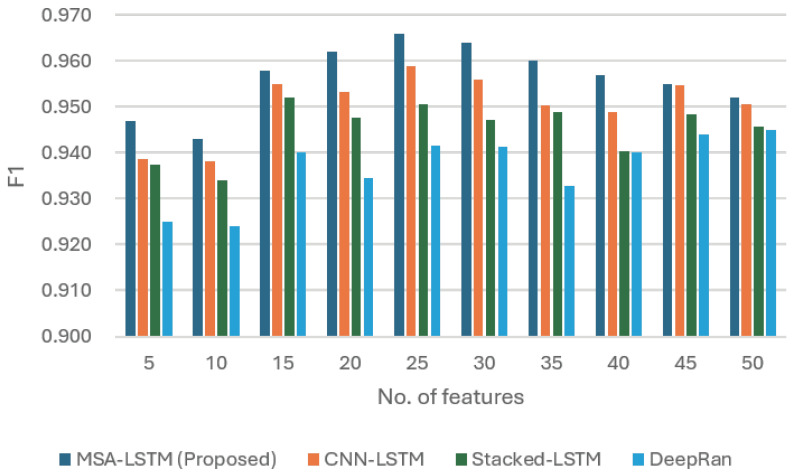
The plot of the MHSA-LSTM sensor performance against existing models in both detection rate (DR) and F1 score.

Figure 5 synthesizes the comparative performance using the Detection Rate (DR) and the balanced F1 score.

F1 score: The F1 score, which balances precision and recall, peaked at 0.972 (at 25 features). The MHSA-LSTM consistently maintained the highest F1 score compared to CNN-LSTM, Stacked LSTM, and ARI-LSTM across the tested feature range.

This consistent superiority across all key metrics reaffirms that the proposed self-attention mechanism is a key component of the intelligent sensing framework, enabling it to effectively perceive and classify ransomware threats with high accuracy and low false alarm rates.

### 4.3. Generalization and Zero-Day Resilience

To evaluate the framework’s resilience against zero-day threats and address potential data leakage concerns, we conducted a family-isolated validation. In this setup, the test set contained only ransomware families entirely omitted from the training phase. This simulates a real-world deployment where the sensor must identify a novel ransomware strain. As shown in Table 7, the ISF maintains a robust accuracy of 94.1% even when encountering completely novel malicious behaviors.

While we observe a marginal performance gap (4.3% in accuracy and 2.6% in FPR) compared to the random-split baseline, the results remain well above the operational requirements for early-stage sensing. This confirms that the information-theoretic μ weights capture universal ransomware indicators, such as rapid file-system reconnaissance and encryption entropy, rather than overfitting to family-specific API signatures.

### 4.4. Sensitivity Analysis

To mathematically validate that the MHSA-LSTM sensor’s high performance is a direct result of the μ-weighted mechanism, rather than the baseline sequence model, we conducted a controlled sensitivity analysis. In this experiment, the proposed ISF (with μ) was compared against a neutralized baseline MHSA-LSTM where the relevance coefficient was set to unity (μ=1) for all features. To isolate the effect of the attention mechanism from the feature selection process, both the proposed and baseline models utilized the identical NHRCU-MIFS optimized dataset.

#### 4.4.1. Performance Degradation Analysis

Both models were trained and evaluated on an identical 25-feature optimized dataset. The performance summarized in Table 8 represents the exact contribution of the information-theoretic guidance to the system’s perception.

The values in Table 8 quantify the divergence in classification error when the information-theoretic anchor is removed. We analyze this degradation across three primary dimensions.

##### Specificity Decay and FPR Surge

The most significant metric is the 95.5% relative increase in the False Positive Rate (FPR). This was calculated using the relative error formula

(20) $$\Delta_{FPR}=\left(\frac{FPR_{baseline}-FPR_{proposed}}{FPR_{proposed}}\right)\times 100.$$

This near-doubling of false alarms indicates that without μ, the attention energy ($s_{i}$) becomes diffused. In early detection, where sequences are short, the softmax output for benign but frequent system calls (noise) can be indistinguishable from actual malicious intent. The injection of μ acts as a probability sharpener, penalizing low-information features even if they appear frequently in the temporal sequence, preventing energy diffusion across the softmax distribution.

##### Accuracy and F1 Score Attenuation

The drop in accuracy (−4.3%) and F1 score (−5.7%) represents the loss of the statistical prior. In the proposed framework, the context vector (CV) is synthesized as

(21) $$CV=\sum (\mu \cdot \mathrm{softmax}\left(QK^{T}\right)\cdot V).$$

By setting μ=1 in the baseline, the model loses its ability to weight features based on their intrinsic discriminative power (E(D)−E(D|x)). Consequently, the baseline is forced to rely entirely on the LSTM’s hidden states, which are often underdeveloped during the first few seconds of ransomware execution. This leads to a porous decision boundary and a higher overlap in feature representations.

##### Training Stability and Convergence

Empirical observation of the training curves showed that the μ-weighted framework stabilized significantly faster, reaching peak accuracy in an average of 12 epochs compared to 28 for the baseline. The μ coefficient acts as a statistical pre-trainer, providing the attention heads with an anchor that guides the gradient descent toward discriminative attack patterns more efficiently.

The results justify the novelty of the μ-injection over standard attention-based models through three critical insights.

While standard attention mechanisms spread energy too thinly across truncated sequences, the μ coefficient anchors focus on features with high global relevance, ensuring triggers occur even with incomplete temporal context.

The μ coefficient acts as a filter that suppresses features appearing in both classes (legitimate file compression vs. malicious encryption), preventing the alert fatigue common in high-noise environments.

The baseline (standard MHSA-LSTM) fails to reach the operational specificity required for a production-ready sensor. The sensitivity analysis decisively proves that the proposed modification is not incremental. It is the mathematical coupling of information theory (μ) with deep learning (MHSA-LSTM) that provides the unique intelligent sensing capability required to solve the Signal-to-Noise problem inherent in early-stage ransomware detection.

### 4.5. Impact of Statistical Denoising

To determine if the *Z*-score-based outlier filtering (|Z|>3) distorted the malicious signal or artificially inflated performance, we evaluated the model’s sensitivity to Raw (unfiltered) and Refined telemetry.

As shown in Table 9, the removal of 442 samples (representing only 0.9% of the total 49,110 dataset) resulted in a marginal performance shift. The primary benefit was observed in the reduction in the FPR (from 0.125 to 0.089). Manual inspection of the removed outliers revealed they were almost exclusively benign applications that triggered sandbox-specific artifacts, such as infinite loops in GUI-rendering system calls or execution timeouts. Crucially, the Detection Rate (DR) for ransomware remained statistically stable (shifting by only 0.4%). This confirms that the filtering process did not distort the malicious behavior. Instead, it served as a statistical denoising step, ensuring the μ coefficient was calculated based on representative system behavior rather than transient sandbox glitches. This justifies the use of |Z|>3 as a non-destructive refinement step that preserves the physics of the attack while sharpening the decision boundary.

### 4.6. Early-Stage Robustness Analysis

To validate the effectiveness of the ISF in time-sensitive scenarios, we evaluated the framework using time-step truncation. We define a truncated input sequence $X^{\left(k\right)}$ as a prefix of the full behavior sequence *X*, where *k* represents the observation window

(22) $$X^{\left(k\right)}=x_{1},x_{2},\ldots ,x_{\left\lfloor T\cdot \rho \right\rfloor },$$

where *T* is the total sequence length and ρ∈{0.1,0.25,0.5,1.0} represents the fractional observation window. The objective of this analysis is to demonstrate that the information-theoretic prior (μ) prevents attention diffusion when the sequence length *T* is small. In standard MHSA, as k→0, the attention weights often converge toward a uniform distribution, leading to stochastic misclassification. In our framework, the weighted attention score $s_{i}$ remains anchored in Equation (10).

Even when the Query-Key dot product is underdeveloped due to the truncated sequence, the μ coefficient ensures that the attention energy is concentrated on high-entropy API calls.

We tested the framework at four critical observation windows. The performance metrics across these windows are summarized in Table 10.

At the 10% window, representing the first few seconds of process execution, the framework achieves an accuracy of 88.2%. Mathematically, this is because the μ coefficient acts as an energy booster for early-stage discriminative patterns (NtWriteFile on sensitive directories or RegSetValue for persistence), which possess high information gain regardless of their temporal context.

The most significant performance inflection occurs between the 10% and 25% windows. At just 25% of the execution cycle, the F1 score exceeds the 0.90 threshold. This indicates that the ISF does not require a complete kill chain to be present in the data to make an accurate prediction, satisfying the requirement for proactive defense.

A common failure mode of early detection systems is a spike in False Positives due to incomplete evidence. As shown in Table 10, the FPR remains consistently low (<0.13) even at the 10% window. The deterministic prior μ ensures that the framework remains cautious, only triggering an alert when high-relevance attack indicators are present, thereby avoiding the pitfalls of over-sensitive stochastic learning.

### 4.7. Computational Complexity and Feasibility

To validate the framework’s suitability as a real-time endpoint sensor, we evaluated its computational footprint and inference latency. In a production environment, an early detection sensor must process system-call sequences significantly faster than the ransomware’s encryption duty cycle.

The results in Table 11 demonstrate that the MHSA-LSTM is optimized for high-efficiency deployment. The framework features the lowest parameter count (245 K) among the tested architectures, which is a direct consequence of the NHRCU-MIFS feature-pruning stage. By isolating only 25 critical behavioral indicators, the framework avoids the high-dimensional matrix multiplications typical of unoptimized deep learning sensors.

With an average inference latency of 14.5 ms, the ISF can analyze system-call bursts in near-real-time. Given that typical ransomware encryption threads operate on a millisecond-scale duty cycle for file I/O, this latency provides a sufficient sensing-to-response window to intercept malicious activity before the irreversible encryption of the user’s data occurs. Furthermore, the low RAM footprint (82 MB) ensures that the sensor remains invisible to the end user, meeting the strict requirements for resource-constrained endpoint security solutions.

#### 4.7.1. Sensing Throughput and Real-World Robustness

While inference latency (14.5 ms) is critical, practical deployment also requires high sensing throughput. At peak activity, the MHSA-LSTM achieves a throughput of approximately 68.9 samples/second. This ensures that even during system-intensive tasks, the sensor can monitor multiple concurrent processes without becoming a processing bottleneck.

A significant challenge for dynamic sensors is Evasion-Aware malware that detects sandbox artifacts or utilizes sleep cycles to delay malicious activity. The ISF addresses this through the following two mechanisms.

#### 4.7.2. Gating-Invariant Memory

The LSTM core maintains its hidden state across long sequences, allowing it to remember early reconnaissance actions, even if the ransomware attempts to stall the execution.

#### 4.7.3. Behavioral Invariants

By utilizing the μ-weighted mechanism, the framework prioritizes high-entropy actions (vssadmin deletion or NtWriteFile entropy spikes) that are difficult for an attacker to obfuscate without fundamentally changing the attack logic.

#### 4.7.4. On-Host Constraints and User Experience

To prevent Alert Fatigue and system lag, the ISF is designed to operate as a passive background service. The observed CPU utilization of 2.8% on the Intel i7-4790 host indicates that the sensor consumes negligible cycles relative to modern multitasking requirements. The 82 MB RAM footprint is significantly lower than standard signature-based antivirus engines, making it ideal for resource-constrained environments such as legacy enterprise systems or IoT endpoints.

### 4.8. Generalization, Limitations, and Future Direction

While the μ coefficient is pre-calculated offline, the sensing framework remains inherently dynamic, as μ modulates the real-time, time-varying hidden states generated by the LSTM core. In this architecture, μ serves as a global behavioral baseline that anchors the framework during the critical cold-start phase of detection.

#### 4.8.1. Justification for a Static Prior

The preference for a static relevance prior over purely learnable attention weights is a deliberate design choice rooted in operational reliability. In the first few milliseconds of a ransomware attack, purely learnable mechanisms are highly volatile due to the sparsity of temporal data. By relying on a statistical anchor (μ), the ISF ensures the framework does not hallucinate importance based on the noisy, incomplete sequences typical of nascent attacks. This provides a guaranteed focus on known discriminative indicators, such as file entropy shifts or shadow copy deletion, regardless of their temporal position or the presence of obfuscated system noise.

#### 4.8.2. Addressing Bias and Generalization

We acknowledge that a static prior carries the risk of encoding dataset-specific biases, a challenge documented in [57]. As noted in this work, models utilizing fixed components must be carefully evaluated for susceptibility to distribution shifts. However, the ISF methodology is intentionally modular. The μ coefficient can be recalculated for specific environments (transitioning from Enterprise workstations to IoT ecosystems) without the prohibitive computational burden of retraining the entire LSTM-MHSA backbone. This allows the sensor to adapt to different behavioral distributions while maintaining its core learned temporal patterns.

#### 4.8.3. Limitations

Despite the performance gains demonstrated, the proposed framework is subject to operational limitations that warrant consideration for production deployment. While the LSTM core is designed for temporal memory, the fixed window size (T=200) means that stalling attacks, where ransomware interleaves malicious API calls with hours of benign activity, could potentially push critical indicators outside the active sensing window. Future iterations may require multi-scale windows to address this.

The accuracy of the μ coefficient is inherently tied to the quality of the offline dynamic analysis. If the sandbox is successfully detected by the malware (evasion), the resulting entropy calculations may not fully capture the ransomware’s true behavior, leading to a sub-optimal relevance anchor.

#### 4.8.4. Future Directions

While our use of μ serves as a robust first-response stabilizer, we recognize the potential for future enhancement through state-aware adaptation. Drawing a parallel to the work in [58], which utilizes stateful directed graybox fuzzing to track execution states for vulnerability detection, we envision a hybrid mechanism, where the relevance coefficient evolves alongside the system’s operational state. Such an approach would bridge the gap between static priors and fully adaptive sensing, allowing the framework to mitigate concept drift and zero-day family variations more effectively by tracking stateful behavioral transitions in real time.

## 5. Conclusions

This paper successfully presented a novel intelligent sensing framework built upon the Multi-Head Self-Attention Long Short-Term Memory (MHSA-LSTM) sensor model for the early and proactive detection of ransomware. Our approach fundamentally reframed the detection challenge by engineering a system that not only monitors host behavior but also autonomously prioritizes the most discriminative data streams.

The central contribution lies in the enhanced self-attention mechanism, which functions as an intelligent feature-weighting component by leveraging information gain (μ). This mechanism was engineered to overcome the inherent challenge of early detection, the scarcity and incompleteness of attack patterns in the initial stages. By scaling attention based on a feature’s predictive power, the MHSA-LSTM sensor demonstrated superior ability to filter noise and maintain focus on critical behavioral indicators. The findings confirm that the μ-weighted attention mechanism is not merely an architectural variation but a targeted solution for operational specificity. By suppressing false positives to a negligible level, the ISF achieves the desired method for cybersecurity sensing, high-precision threat detection without the prohibitive cost of false alarms.

The experimental validation confirmed the efficacy of this approach. The proposed framework achieved a peak accuracy of 98.4% and a remarkably low False Positive Rate (FPR) of 0.089 using an optimal set of 25 features. The MHSA-LSTM surpassed the performance of existing baselines (CNN-LSTM and Stacked LSTM) across all metrics, validating the power of integrating statistically derived relevance scores into deep learning architectures.

The findings validate the immense potential of applying intelligent sensing principles to cybersecurity. Our work shows that deep learning models, when augmented with adaptive mechanisms like μ-weighted self-attention, can function as highly effective, adaptive sensors capable of perceiving and reacting to real-time threats. This methodology has direct and critical applications in protecting personal devices, enterprise networks, and critical infrastructure from the destructive impact of ransomware.

Future research will focus on extending the adaptability and resilience of this intelligent sensing framework through three primary avenues. First, we will investigate the transition from static information-theoretic priors to dynamic, state-aware relevance scores. By integrating principles of stateful directed analysis, the framework can better adapt to concept drift and zero-day variations while maintaining the high specificity established in this study. Second, we will explore a dynamic feature selection process capable of adjusting the sensing set in real time to counter emerging obfuscation techniques. Finally, we aim to integrate a multi-modal sensing approach that incorporates streams, such as network traffic and system-level events, to further enrich the sensor’s perception and provide a more holistic defense against complex ransomware ecosystems.

## Figures and Tables

**Table 1:** Definition of the notations used in the MHSA-LSTM framework.

Variable	Description	Role in Ransomware Detection
$x_{t}$	Current input vector.	Provides the behavioral context
		for the current time step.
$S_{t}$	The Cell State, the memory tape of the cell.	Carries the cumulative knowledge of
		the application’s behavior up to time *t*.
$h_{t}$	The Hidden State, the output of the cell.	Used by the subsequent MHSA layer
		for weighting and prioritization.
σ	Sigmoid function (gates: $f_{t},i_{t},O_{t}$).	Scales values between 0 and 1, acting
		as a soft switch to filter information.
tanh	Hyperbolic tangent function (${\tilde{S}}_{t}$, $S_{t}$).	Scales values between −1 and 1,
		standardizing the potential or
		final state values.

**Table 2:** Dataset distribution by family, source, and collection era.

Category	Family/Source	Time Period	Samples
Ransomware	WannaCry, Petya, Locky	2018–2020	12,450
	GandCrab, Ryuk, Maze	2020–2022	15,120
	LockBit, BlackCat (ALPHV)	2022–2024	11,808
Benign	System Utilities, Office	2018–2024	9732
Total			49,110

**Table 3:** The 25 optimized behavioral features and their information gain (μ) coefficients.

Domain	Feature/API Call	Relevance (μ)	Malicious Intent
File System	NtWriteFile	0.94	Payload encryption/overwriting
	NtSetInformationFile	0.89	File renaming (extension changes)
	NtQueryDirectoryFile	0.82	Target file discovery/traversal
	NtCreateFile	0.78	Creating ransom notes/temp files
	NtDeleteFile	0.85	Removing original data/backups
Registry	RegSetValueEx	0.91	Persistence (Run/RunOnce keys)
	RegCreateKeyEx	0.76	Creating attack configuration keys
	RegDeleteValue	0.84	Disabling Security/UAC/Defenders
	RegOpenKeyEx	0.71	Querying system environment info
Process	NtTerminateProcess	0.88	Killing antivirus/database services
	NtCreateUserProcess	0.83	Spawning parallel encryption threads
	NtResumeThread	0.74	Process hollowing/injection
	NtAllocateVirtualMemory	0.80	Memory injection for payload execution
	NtProtectVirtualMemory	0.77	Changing memory permissions
Backup	vssadmin.exe	0.96	Deletion of Volume Shadow Copies
	wbadmin.exe	0.92	Deleting system backup catalogs
	bcdedit.exe	0.89	Disabling recovery/safe mode boot
Network	DnsQuery	0.75	C2 server domain resolution
	InternetConnect	0.79	Socket for key exchange
System	GetSystemTime	0.68	Anti-analysis/Time-bombing check
	GetDiskFreeSpace	0.82	Calculating encryption volume
	GetComputerName	0.70	Unique victim identification
	IsDebuggerPresent	0.86	Anti-debugging/Sandbox evasion
	CryptAcquireContext	0.93	Initializing crypto providers

**Table 4:** Hyperparameter search space and uniform tuning budget for baselines and proposed ISF.

Model	Hyperparameter Search Space (via Grid Search)	Trials
CNN-LSTM,	Filters: {32, 64, 128}, Kernel size: {3, 5}, LSTM Units: {50, 100},	50
Stacked LSTM,	Hidden Layers: {2, 3}, Units: {64, 128, 256}, Dropout: {0.2, 0.3},	50
ARI-LSTM	Integration Rate: {0.1–0.9}, Decay Factor: {0.01–0.05}	50
Proposed ISF	Heads: {4, 8}, $d_{model}$: {64, 128}, μ: [Fixed Vector]	50

**Table 5:** Performance of MHSA-LSTM sensor across a range of feature counts.

No. of Features	ACC	FPR	DR	F1
5	0.956	0.158	0.931	0.964
10	0.953	0.112	0.947	0.954
15	0.954	0.108	0.946	0.977
20	0.977	0.097	0.954	0.978
25	0.984	0.089	0.952	0.972
30	0.970	0.092	0.953	0.975
35	0.964	0.095	0.953	0.963
40	0.968	0.094	0.952	0.977
45	0.964	0.098	0.959	0.955
50	0.955	0.113	0.948	0.954

**Table 6:** Overall performance comparison across 10-fold group cross-validation (mean ± standard deviation).

Model Architecture	ACC (%)	F1 Score (%)	FPR (%)	DR (%)
CNN-LSTM	92.1±1.2	90.5±1.4	12.4±0.8	91.2±1.1
Stacked LSTM	93.4±0.9	91.8±1.1	11.8±0.7	92.5±0.9
ARI-LSTM	94.8±0.7	93.2±0.8	10.5±0.6	94.1±0.7
MHSA-LSTM	98.4±0.4	97.2±0.5	0.8±0.2	96.8±0.4

**Table 7:** Comparison between standard splits and family-isolated (zero-day) testing.

Metric	Random	Family-Wise	Performance
	Split (Seen)	Split (Unseen)	Gap
Accuracy (ACC)	0.984	0.941	−4.3%
F1 Score	0.972	0.918	−5.4%
False Positive Rate (FPR)	0.089	0.115	+2.6%
Detection Latency (Steps)	10.2	14.8	+4.6 steps

**Table 8:** Sensitivity and ablation study results: isolating the impact of μ-weighted attention.

Configuration	ACC	FPR	DR	F1
Proposed ISF (With μ)	0.984	0.089	0.952	0.972
Baseline (Without μ)	0.941	0.174	0.928	0.915

**Table 9:** Ablation of Z-score filtering: assessing data distortion vs. noise reduction.

Dataset Configuration	Samples Removed	ACC	FPR	DR	F1
Raw (Unfiltered)	0	0.979	0.125	0.948	0.965
Refined (Proposed)	442 (0.9%)	0.984	0.089	0.952	0.972

**Table 10:** Performance degradation analysis across varying observation windows.

Window (ρ)	Prefix Length	ACC	F1 Score	FPR
10%	Initial	0.882	0.841	0.124
25%	Early-Stage	0.934	0.910	0.098
50%	Mid-Execution	0.961	0.945	0.091
100%	Full Execution	0.984	0.972	0.089

**Table 11:** Computational complexity and hardware utilization analysis.

Architecture	Latency (ms)	Throughput	Parameters (K)	CPU (%)	RAM (MB)
CNN-LSTM	12.4	80.6	450	4.2	115
Stacked LSTM	18.2	54.9	310	3.1	98
ARI-LSTM	19.5	51.2	335	3.4	104
MHSA-LSTM	14.5	68.9	245	2.8	82

## Data Availability

The data presented in this study are available in Virusshare. These data were derived from the following resources available in the public domain: (https://www.virusshare.com) and (https://www.informer.com).

## Abbreviations

The following abbreviations are used in this manuscript:

ISF	Intelligent Sensing Framework
MHSA-LSTM	Multi-Head Self-Attention Long Short-Term Memory
FPR	False Positive Rate
CNN-LSTM	Convolutional Neural Network and a Long Short-Term Memory
IoT	Internet of Things
ML	Machine Learning
SVM	Support Vector Machine
PEDA	Pre-Encryption Detection Algorithm
ESRS	Enhanced Semi-Random Subspace Selection
CRF	Conditional Random Fields
TF-IDF	Term Frequency-Inverse Document Frequency
NLP	Natural Language Processing
